# Phenotype and imaging features associated with APP duplications

**DOI:** 10.1186/s13195-023-01172-2

**Published:** 2023-05-11

**Authors:** Lou Grangeon, Camille Charbonnier, Aline Zarea, Stephane Rousseau, Anne Rovelet-Lecrux, David Bendetowicz, Marion Lemaitre, Cécile Malrain, Muriel Quillard-Muraine, Kevin Cassinari, David Maltete, Jeremie Pariente, Olivier Moreaud, Eloi Magnin, Benjamin Cretin, Marie-Anne Mackowiak, Adeline Rollin Sillaire, Martine Vercelletto, Elsa Dionet, Olivier Felician, Pauline Rod-Olivieri, Catherine Thomas-Antérion, Gaelle Godeneche, Mathilde Sauvée, Leslie Cartz-Piver, Isabelle Le Ber, Valérie Chauvire, Therèse Jonveaux, Anna-Chloé Balageas, Annie Laquerriere, Charles Duyckaerts, Anne Vital, Andre Maues de Paula, David Meyronet, Lucie Guyant-Marechal, Didier Hannequin, Elisabeth Tournier-Lasserve, Dominique Campion, CNR-MAJ collaborators, Gaël Nicolas, David Wallon

**Affiliations:** 1 Department of Neurology and CNR-MAJ, Univ Rouen Normandie, U1245 and CHU Rouen, 76000 Rouen, France; 2 Department of Neurology, Rouen University Hospital, Rouen Cedex, 76031 France; 3 Department of Genetics and CNR-MAJ, Univ Rouen Normandie, U1245 and CHU Rouen, 76000 Rouen, France; 4 Neurology Department, Sorbonne Université, Paris Brain Institute – ICM, Inserm, CNRS and APHP, Hôpital de la Pitié-Salpétrière APHP, Paris, France; 5 Geriatric department, Seclin-Carvin Hospital, Seclin, France; 6 Department of Neurology, Rennes Hospital, Rennes, France; 7 Laboratoire de biochimie, Rouen University Hospital and University of Rouen, Rouen, France; 8 Neurology Department, Toulouse University Hospital and Toulouse NeuroImaging Center (ToNIC) INSERM-Univeristy of Toulouse Paul Sabatier, Toulouse, France; 9 Department of Neurology, Grenoble Hospital, Grenoble, France; 10 Department of Neurology, Besancon Hospital, Besancon, France; 11 Department of Neurology, Hautepierre Hospital, Strasbourg, France; 12 Univ. Lille, CHU Lille, CNRMAJ, 59000 Lille, France; 13 Department of Neurology, Nantes University Hospital, Nantes, France; 14 Department of Neurology, Clermont-Ferrand Hospital, Clermont-Ferrand, France; 15 APHM, Service de Neurologie et Neuropsychologie, CHU Timone, Marseille, France; 16 Aix Marseille Univ, INSERM, INS, Inst Neurosci Syst, Marseille, France; 17 Neurology Department, Hôpital Saint Anne APHP, Paris, France; 18 Department of Neurology, Lyon University Hospital, Lyon, France; 19 Department of Neurology, La Rochelle Hospital, La Rochelle, France; 20 Centre Mémoire Ressources et Recherche (CMRR), Limoges University Hospital, Limoges, France; 21 Department of Neurology, Angers University Hospital, Angers, France; 22 Department of Neurology, Nancy University Hospital, Nancy, France; 23 CHRU Tours, Centre Mémoire Ressources et Recherche (CMRR), Tours, France; 24 Department of Neuropathology, F 76000, Normandy Center for Genomic and Personalized Medicine, Normandie Univ, UNIROUEN, Inserm U1245 and Rouen University Hospital, Rouen, France; 25 Sorbonne Unviersité, INSERM, CNRS U1127, ICM and Laboratoire de Neuropathologie R. Escourolle, Hospital Pitie-Salpêtrière, Paris, France; 26 Department of Pathology, University Hospital, Bordeaux, France; 27 Department of Pathology, La Timone University Hospital, Marseille, France; 28 Department of Pathology, Hopital Civil University Hospital, Lyon, France; 29 AP-HP, Groupe Hospitalier Saint-Louis Lariboisière-Fernand-Widal, Service de Génétique Moléculaire Neurovasculaire, INSERM UMR 1141, NeuroDiderot, Université de Paris, Paris, France

**Keywords:** Cerebral amyloid angiopathy, Alzheimer disease, APP duplication, Cerebral MRI, Autosomal dominant

## Abstract

**Background:**

*APP* duplication is a rare genetic cause of Alzheimer disease and cerebral amyloid angiopathy (CAA). We aimed to evaluate the phenotypes of *APP* duplications carriers.

**Methods:**

Clinical, radiological, and neuropathological features of 43 *APP* duplication carriers from 24 French families were retrospectively analyzed, and MRI features and cerebrospinal fluid (CSF) biomarkers were compared to 40 *APP*-negative CAA controls.

**Results:**

Major neurocognitive disorders were found in 90.2% symptomatic *APP* duplication carriers, with prominent behavioral impairment in 9.7%. Symptomatic intracerebral hemorrhages were reported in 29.2% and seizures in 51.2%. CSF Aβ42 levels were abnormal in 18/19 patients and 14/19 patients fulfilled MRI radiological criteria for CAA, while only 5 displayed no hemorrhagic features. We found no correlation between CAA radiological signs and duplication size. Compared to CAA controls, *APP* duplication carriers showed less disseminated cortical superficial siderosis (0% vs 37.5%, *p* = 0.004 adjusted for the delay between symptoms onset and MRI). Deep microbleeds were found in two *APP* duplication carriers. In addition to neurofibrillary tangles and senile plaques, CAA was diffuse and severe with thickening of leptomeningeal vessels in all 9 autopsies. Lewy bodies were found in substantia nigra, locus coeruleus, and cortical structures of 2/9 patients, and one presented vascular amyloid deposits in basal ganglia.

**Discussion:**

Phenotypes associated with *APP* duplications were heterogeneous with different clinical presentations including dementia, hemorrhage, and seizure and different radiological presentations, even within families. No apparent correlation with duplication size was found. Amyloid burden was severe and widely extended to cerebral vessels as suggested by hemorrhagic features on MRI and neuropathological data, making *APP* duplication an interesting model of CAA.

**Supplementary Information:**

The online version contains supplementary material available at 10.1186/s13195-023-01172-2.

## Introduction

Cerebral amyloid angiopathy (CAA) is mainly characterized by pathological deposition of Amyloid-β (Aβ) peptides in the walls of cortical and leptomeningeal vessels. CAA may lead to intracerebral hemorrhages such as microbleeds (CMB), hematomas (ICH), focal subarachnoid hemorrhage, and cerebral superficial siderosis (CSS). In cerebral autopsy series, Alzheimer disease (AD) is frequently associated with some degree of CAA [1]. AD is neuropathologically defined by an abnormal aggregation of Aβ peptides in the brain parenchyma, along with neurofibrillary tangles composed of intra-neuronal abnormally hyperphosphorylated Tau proteins. Aβ peptides thus play a central role in both CAA and AD [1]. Despite the existence of other peptides putatively causing CAA in rare genetic forms, Aβ aggregation is the major peptide responsible for CAA in elderly people and most CAA patients.

Among AD patients, 4 to 10% exhibit the first symptoms before the age of 65 defining early-onset AD (EOAD) [2–4]. In this population, a monogenic cause is identified in ~2 to 77%, depending on ages at onset and family history [5–8]. Such pathogenic variants in either the amyloid-β protein precursor (*APP*), presenilin 1 (*PSEN1*), or presenilin 2 (*PSEN2*) genes or duplications of the *APP* locus are autosomal dominantly inherited. After their discovery in 2006 [9], *APP* locus duplications were described in autosomal dominant EOAD series with various frequencies, 8% (95% CI, 2.6–17.1) in France [9] and 2.7% (95% CI, 0.32–9.3) in the Netherlands [10]. This copy number variant (CNV), located on chromosome 21, may encompass the *APP* gene with or without surrounding genes. However, no correlation has been identified between the phenotype of *APP* duplication carriers and the size of the duplicated locus or the genes included but the small number of reported families precluded any definite conclusion [11]. *APP* is clearly the main gene as its duplication is sufficient to cause EOAD and/or CAA through overexpression. mRNA levels are increased ~1.5 times in carriers [9, 12] with widespread Aβ deposits in the brain parenchyma and vessels [13]. Recently, we reported a family with an *APP* triplication (4 copies of *APP*) and a phenotype similar to *APP* duplications [14].

Some degree of inter and intrafamilial phenotype diversity has been reported with *APP* duplication but from a small number of families [11, 15]. Most carriers presented AD-related cognitive decline and around 30% with CAA-related lobar spontaneous ICH upon presentation. Moreover, *APP* duplication carriers were more likely to present seizures [16]. Neuropsychiatric disorders with hallucinations related to a pathologically proven Lewy body (LB) disease have also been reported [15]. Given the rarity of *APP* duplications, little is known of the radiological pattern. Large discrepancies have been reported, from normal neuroimaging to severe CAA with multiple CMB or large inflammatory-related CAA [17]. The proportion of *APP* duplication carriers exhibiting CAA according to Boston-imaging criteria [18] (except the age criterion) remains unknown and studies on AD cerebrospinal fluid (CSF) biomarkers are still required.

Here, we analyzed the clinical, radiological, and neuropathological features of 43 patients carrying 24 distinct *APP* duplications and compared their MRI features to 40 *APP*-negative CAA controls.

## Material and methods

This retrospective study analyzed the phenotypic data of *APP* duplication carriers detected in France in the CNRMAJ center (Rouen University Hospital) from a nation-wide recruitment since 2006. Patients or legal representatives provided informed written consent for genetic analyses, in a medical and research setting (RBM 02-59, EudraCT 2009-010884-18) or GMAJ, NCT01622894, respectively approved by Paris Ile de France II and CPP Nord Ouest I ethics committees. This study was also conducted in accordance with the Declaration of Helsinki.

### Inclusion and genetic analyses

The CNRMAJ laboratory of Rouen has a nation-wide recruitment of blood samples from patients with EOAD and/or with early-onset CAA (onset before 66 years). DNA isolated from whole blood samples of EOAD ± CAA were screened by Sanger or exome sequencing for exons 16-17 of *APP*, *PSEN1*, and *PSEN2* coding exons and for *APP* duplication by quantitative multiplex PCR of short fluorescent fragments (QMPSF), as previously described [19], and CAA patients (without AD phenotype) were screened for *APP* pathogenic variants and duplications. Genes surrounding *APP* were assessed by additional QMPSF or digital droplet PCR [20] experiments in order to assess the size of each duplication and its gene content. All patients were genotyped for *APOE* by Sanger sequencing. Patients were recruited regardless of the presence of a positive family history.

We included all patients exhibiting *APP* locus duplication including probands and their relatives. Before diagnosis, patients had neurological examination, neuropsychological assessment, and some had cerebral MRI and CSF AD biomarker analysis. Clinical data were retrieved from patients’ medical charts provided by each referring clinician.

The MRI patterns of these patients were compared to a control group of 40 *APP*-negative CAA controls (by sequencing exons 16 and 17 and by QMPSF) referred either to CNRMAJ, Rouen or to the Department of Genetics, Lariboisière Hospital, Paris, and fulfilling radiological criteria for probable CAA [18].

### MRI analysis

All cerebral 1.5 or 3-T MRI containing magnetic-susceptibility sequences were assessed. These blood-sensitive sequences, used to evaluate intracranial bleeds, were T2 gradient echo (T2 GRE) sequences, T2*, susceptibility weighted imaging (SWI), or a T2*-weighted angiography (SWAN), depending on the MRI machine and protocol used. The diagnosis of probable CAA was performed according to revised Boston diagnostic criteria, except for the age criterion (> 55 years) [18]. In order to assess a consensus, two clinicians (LG and DW) independently rated hemorrhagic features in each brain region of interest. All hemorrhagic lesions were analyzed independently by both experts, by visually counting each lesion. The presence and number of CMBs (< 10 mm diameter) and ICH (≥ 10 mm) was evaluated [21], and CSS was classified as focal (≤ 3 sulci) or disseminated (> 4 sulci) after recording the number of sulci involved [22]. Hippocampal atrophy was scored according to Scheltens scale on 3D acquisition or 2D coronal T1-weighted-sequences [23]. Perivascular white matter lesions were scored according to Fazekas scale on T2 or FLAIR-weighted sequences [24]. Lesions were also classified according to location: lobar pre- or post-rolandic, deep, or in posterior fossa. In case of repeated MRI, only the latest images were considered for rating.

### CSF analysis

CSF was obtained by lumbar puncture (LP). All centers used a common 10-ml polypropylene tube to collect CSF (catalog number 62.610.201; Sarstedt, Nümbrecht, Germany). All samples were aliquoted after centrifugation for 10 min at + 4 °C in polypropylene Eppendorf tubes and then frozen at – 80 °C within 1 h. Aβ42, Aβ40, Tau, and p-Tau protein measurements were taken using an enzyme-linked immunosorbent assay (ELISA) (Fujirebio-Europe, Ghent, Belgium) according to the manufacturer’s instructions. Analysis was performed in duplicate and a coefficient of variation (CV) less than 15% was considered as acceptable. In this case, the mean of the two measured values was taken as final result. All sites belong to the same national ePLM network which was created to enhance harmonization of procedures regarding CSF AD biomarkers [25]. As preanalytical and analytical procedures might still have impact on the quantification, we set the normal thresholds for all CSF biomarkers following local laboratory normal ranges.

### Neuropathological examination

Nine brain autopsies were available (BES_262, EXT_298, EXT_773, EXT_144, EXT_019, two patients from ROU_037 and two patients from EXT_028). The brains were fixed in a 10% formaldehyde solution buffer for at least 3 months. Seven-micrometer sections were cut from paraffin-embedded blocks of frontal, temporal including hippocampus, occipital lobes, and cerebellar hemispheres and brainstem. Sections were stained with hematoxylin–eosin, periodic acid Schiff, Orcein, Luxol-Phloxine. Routine immunohistochemical study was performed using antibodies directed against alpha-synuclein (diluted 1/200) (Zymed, Clinisciences, Montrouge, France), PHF tau (AT8, 1/20) (Innogenetics, Gent, Belgium), glial fibrillary acidic protein (GFAP, 1/300), PrP (1/50), ubiquitin (1/100), and the macrophagic marker CD68 (1/300) (Dakopatts, Trappes, France). Vascular and intraparenchymatous amyloid deposits were characterized using β-amyloid protein antibody (diluted 1/100) (Dakopatts, Trappes, France).

### Statistical analyses

Results are expressed as mean ± standard deviation (SD) unless otherwise specified. Fisher exact tests and Welch two-sample tests were used to compare radiological characteristics between *APP* duplication carriers and the comparison CAA group. Point estimates of odds-ratios and mean differences between the two groups were accompanied by corresponding 95% confidence intervals, as provided by the R fisher.test and t.test functions, without continuity correction. Logistic regression was used with adjustment for time from symptoms onset to MRI. Exception was made for disseminated sulci. Because none of APP duplication carriers displayed disseminated loci, logistic regression could not be used. Adjusted OR and *p*-value were computed using Firth logistic regression with the logistf R package using default penalization parameters We analyzed radiological findings using the R statistical software version 3.6.2.

### Data availability statement

De-identified database and statistical analysis plan will be shared upon reasonable request for 2 years after publication.

## Results

### *APP* duplication families

**Fig. 1:**
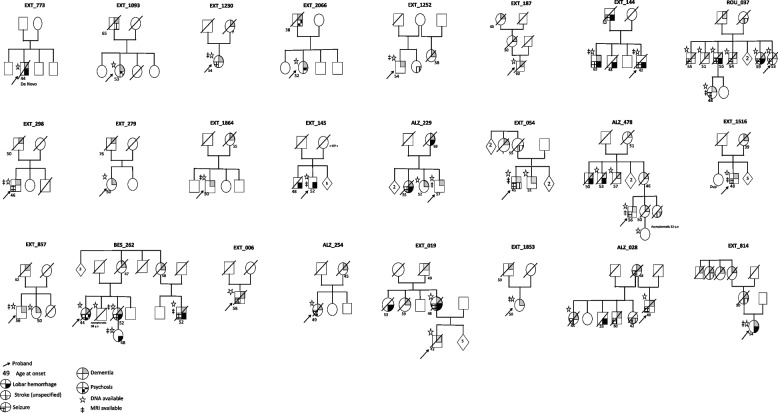
Reduced pedigrees of 24 APP duplication families

Overall, 43 *APP* duplication carriers, 41 symptomatic and 2 presymptomatic, from 24 families were included (Table 1). Age of onset was observed from 42 to 63 years. For 21 patients, age at death ranged from 42 to 68 years (mean: 58.6 years), occurring after mean disease duration of 9.33 ± 6.3 years. All patients had a positive family history except one who displayed a de novo occurrence, already reported in 2015 [26]. The two presymptomatic individuals did not present any symptoms at the age of 32 and 38 years, respectively (Fig. 1).

1. Genetic results for APP and APOE genes

**Table 1:** All 24 French families carrying an *APP* duplication

ID fam	Duplication size (Mb) (Hg19)	AAO (years)	DD of sampled patients (years)	***N*** patients sampled	***N*** affected or highly suspected affected members
EXT_006	15.3	58	3	1	NA
EXT_019	3.6	42-59	12	2	5
ALZ_028	0.6	42-60	15-30	2	6
ROU_037	0.75	48-59	5-12	7	8
EXT_054	11	45-55	1-12	2	4
EXT_144	9.7	42-63	1-4	2	4
EXT_145	6.1	48-52	6	1	3
EXT_187	14.2	45-60	4	1	3
ALZ_229	6.1	52-69	1-18	2	4
ALZ_254	14.7	45-49	6	1	2
BES_262	0.55	44-58	5-32	5	8
EXT_279	1.08	50 -76	3	1	2
EXT_298	0.78	46-50	13	1	2
ALZ_478	1.9	46-57	3-9	4	8
EXT_773	6.3	44	12	1	1 (de novo)
EXT_814	5.7	50-54	8-10	1	6
EXT_857	1.5	50-62	1-2	2	3
EXT_1093	1.5	53-65	10	1	2
EXT_1230	9.3	54	7	1	2
EXT_1252	1.4	54-58	8	1	3
EXT_1516	0.95	39-48	4	1	2
EXT_1853	0.95	50	7	1	2
EXT_1864	3.4	50-55	6	1	2
EXT_2066	0.6	38-52	2	1	2

*ID Fam* family identification number, *AAO* age at onset, *DD* disease duration, i.e., time from symptoms to death or last evaluation, *N* number

The duplicated segments had a minimal size ranging from 0.55 to 15.3 Mb and contained 3 to 25 protein-coding genes; all duplications encompassed at least the *APP* gene entirely (Fig. 3). Retrospective analysis of medical records revealed no clinical features suggestive of Down syndrome in any patient, as expected, as the critical Down syndrome region on chromosome 21 was never encompassed in the duplicated segment. Regarding the *APOE* genotypes, three (7.3%) patients carried at least one *APOE* ε2 allele (*APOE2*) and six (14.6%) carried at least one *APOE* ε4 allele (*APOE4*) (no homozygous) (Table 2). According to cox proportional hazard models, no association was found between age at onset or disease duration and sex (*p* = 0.34 and 0.74 respectively) or *APOE* genotype (*p* = 0.63 and 0.11 respectively). No correlation was found either between age at onset and the duplication size (*p* = 0.15 and 0.66 respectively). If we individually look at each duplicated surrounding genes, no statistical significance was found either (lowest *p* value = 0.13 for NCAM2 or TUBAP).

2) Clinical spectrum in APP duplication carriers

**Table 2:** Demographics and clinical characteristics of symptomatic APP duplication carriers (*n* = 41)

**Age at onset,** years, mean ± SD	50.8 ± 5.9
**Age at death,** years, mean ± SD	58.6 ± 6.2 *(n = 21)*
**Sex, male** (*n* %)	25 (60.9%)
**First neurologic event**	
**ICH, ***n* patients (%)	5 (12.1%)
**Cognitive decline, ***n* patients (%)	32 (78.0%)
**Ischemic stroke, ***n* patients (%)	0
**Psychiatric symptoms ***n* patients (%)	2 (4.9%)
**Seizures, ***n* patients (%)	2 (4.9%)
**Symptomatic lobar ICH**	12 (29.2%)
**Age at 1st ICH (years, mean** ± SD**)**	47.2 ± 18.8
**Seizure, *n *patients (%)**	21 (51.2%)
**Presence of cognitive decline, *n *patients (%)**	37 (90.2%)
***APOE*genotype**	
*APOE2* carriers *n* (%)	3 (7.3%)
*APOE4* carriers *n* (%)	6 (14.6%)
*APOE2 or 4* homozygous *n* (%)	0
**CSF biomarkers available,*n*(%)**	19 (46.3%)
Aβ-42 (pg/ml), mean ± SD	378.1 ± 133.1
Tau (pg/ml), mean ± SD	600.5 ± 318.0
Phospho-Tau (pg/ml), mean ± SD	84.0 ± 39.3

*ICH* intra cerebral hemorrhage

**Fig. 2:**
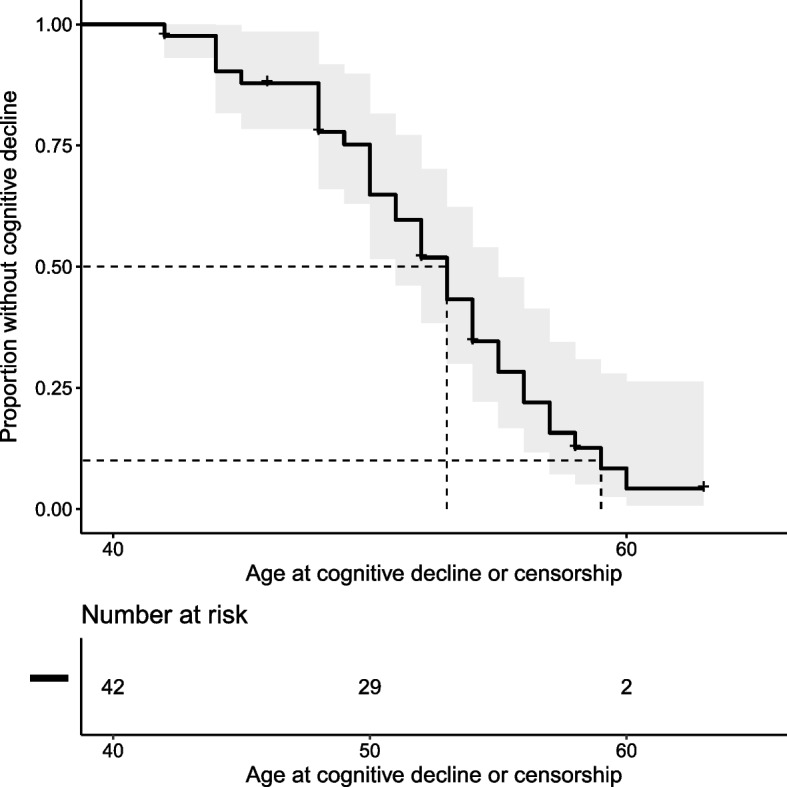
Kaplan-Meier curve showing cognitive decline over time. In the cox-proportional hazard model, the two individuals without complaint were censored. At 52 of age, 48.1% [29,2–71.6%] of patients suffer from cognitive decline. At 59 of age, 91.6% [72,1–97.5%] of patients suffer from cognitive decline

Cognitive impairment was observed from 42 to 60 years (mean: 50.7 years) in all but four (90.2%). Seventeen patients (41.4%) presented amnestic syndrome of the hippocampal type suggestive of AD, four (9.7%) with a mainly behavioral presentation. Nine patients (21.9%) had a rapid progression and quickly became bedridden (i.e., within 5 years after symptoms onset), which prevented further neuropsychological classification. Two patients (4.8%) presented moderate attention impairment after ICH, one (2.4%) with a clinical diagnosis of LB disease. No further details were available for six patients (14.6%). Five patients (12.1%) presented atypical presentations. Two developed psychiatric disorders with visual hallucinations associated with bilateral tremor, suggestive of LB disease; another had initial psychiatric disorders with dissociative delirium followed by severe cognitive decline within 5 years. Finally, two patients developed social or eating behavioral disorders with cognitive decline leading in line with a frontal variant of AD, quickly followed by repeated seizures and a bedridden state. The Kaplan-Meier curve showed onset of cognitive decline before 59 in 90% of cases (Fig. 2). Symptomatic ICH was reported in twelve patients (29.2%), from 42 to 63 years (mean: 46.7 years). Seizures occurred in 21 (51.2%) patients: 11 in the first 5 years of cognitive decline or before. One patient presented seizures and an aspect of CAA-related inflammation on MRI according to current diagnostic criteria [27].

3) CSF biomarkers

Nineteen *APP* duplication carriers underwent LP with measurement of AD CSF biomarkers. Aβ42 levels were below the normal threshold in all but one. Phospho-Tau levels were over the normal threshold in 15/19 patients (78.9%), and total Tau protein levels were over the normal threshold in 11/19 (57.8%). Overall, all patients with a clinical diagnosis of AD showed abnormal levels of at least two out of three CSF biomarkers, whereas four (26.3%) patients showed normal phospho-Tau and total Tau levels. None of the four patients with isolated decreased Aβ42 levels presented a cognitive decline typical of AD. The mean ratio phospho-Tau/Ab42 was 0.25 ± 0.16 and 18/19 had a ratio over the pathological threshold of 0.11 according to Welge et al. [27].

### Neuroimaging in APP duplication families

1. Hemorrhagic imaging features

MRI with blood-sensitive sequences was available for 19 patients. Five carriers showed no hemorrhagic features on MRI (mean time between symptom onset and MRI 3.9 years ± 2.2), whereas 14 (73.6%) fulfilled the revised Boston imaging criteria for probable CAA diagnosis (mean time between symptom onset and MRI 4.0 years ± 3.1). Four carriers showed no ICH but multiple CMBs. Two *APP* duplication carriers showed focal CSS (no disseminated CSS). Overall, the numbers of CMBs ranged from 0 to 420 and the numbers of ICH from 0 to 3. Surprisingly, two *APP* duplication carriers showed one CMB in the basal ganglia (Supp Figure 1). Of note, one of these two patients had no history of hypertension and the other one was given antihypertensive medication after ICH occurrence, without available data regarding this condition before.

2) Genotype-imaging correlation

**Fig. 3:**
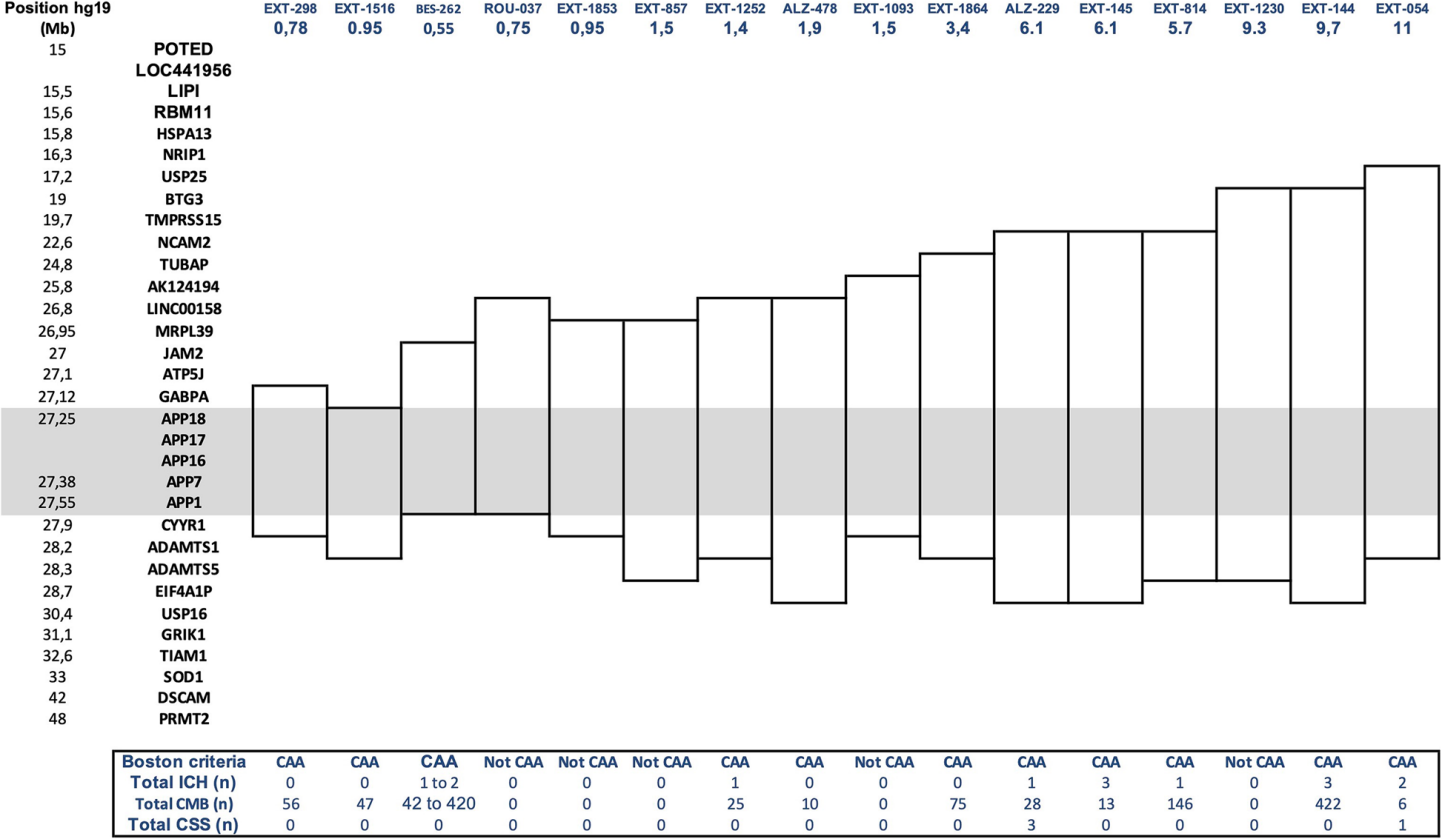
Size of APP locus duplications (*n* = 16) for each carrier with MRI available and associated genes involved. Rectangles show the duplicated regions in each family. For the family BES-262, MRIs of three related patients were available, and therefore, the number of ICH or CMBs varied between individuals. Otherwise, only one MRI was available per family. First column shows the position on chromosome 21 (GRCh37); second column shows name of genes corresponding to the position or the exon number within the APP gene; following columns represent the genomic duplication carried by each family (with family number and size of duplication in Mb). Each duplication contains the complete grey line corresponding to the APP gene. CAA, cerebral amyloid angiopathy diagnosis on MRI according to Boston revised criteria (except age criterion); ICH, intracerebral hemorrhage; CMB, cerebral microbleed; CSS, cortical superficial siderosis. Intra-familial heterogeneity was observed in the BES_262 family, in which 3 MRIs of symptomatic patients were available. The number of CMBs ranged from 42 to 420 (MRI performed at 54 and 56 years of age, respectively), and one patient from this family showed CAA-related inflammation at 52 years of age [17]

No correlation was observed between CAA radiological signs and *APP* duplication size (Fig. 3). For instance, patients carrying smaller duplications (size < 1 Mb) could show from no CMBs (0.75Mb in ROU_037 at age 49) up to 420 CMBs (0.55Mb in BES_262 at age 56). No correlation was observed with the other genes included in the duplication. No association was found between age at onset and presence of ICH (*p* = 0.77) or total number of microbleeds (*p* = 0.29), neither between CSF Aβ42 levels and MRI features (data not shown).

Intrafamilial heterogeneity was observed in the BES_262 family, in which 3 MRIs of symptomatic patients were available (Supp Figure 2). The number of CMBs ranged from 42 to 420 (MRI performed at 54 and 56 years of age, respectively), and one patient from this family showed CAA-related inflammation at 52 years of age [17]. Among the 14 patients fulfilling the revised Boston imaging criteria for probable CAA diagnosis, two patients from the same family (EXT_144) carried one *APOE*2 allele, one (ALZ_478) carried an ε2/ε4 genotype and the other (EXT_298) carried one *APOE*4 allele. Conversely, the five patients with no hemorrhagic features on MRI were *APOE*3 homozygous.

### Neuroimaging comparison of APP duplication carriers and CAA controls

CAA controls (*n* = 40) had a mean age at onset of 63.0 ± 9.7 years. Initially, 11 (27.5%) had cognitive decline and 22 (55.0%) had ICH. The remaining patients (*n* = 7) presented seizures (*n* = 3), transient focal neurological episodes (TFNE) (*n* = 2), or cephalalgia (*n* = 2). Overall, eleven controls (27.5%) had seizures during the disease course. Fifteen (37.5%) carried at least one *APOE*4 allele and 7 (17.5%, not homozygous) carried at least one *APOE*2 allele (not homozygous). The radiological features of these controls were compared to those of the *APP* duplication carriers with available MRI and who fulfilled Boston criteria for CAA (*n* = 14). *APP* duplication carriers seemed to show a higher number of total CMBs compared to CAA controls (mean 110.7 vs 56.7) with a prominent posterior location of CMBs (mean posterior /anterior ratio of 10.1 vs 5.3 respectively), even though not statistically significant. Conversely, CAA controls showed an elevated rate of CSS (52.5% vs 14.2%, *p* < 0.02) and more frequently disseminated than *APP* duplication patients (37.5% vs 0%; *p* = 0.005) Anterior white matter lesions were more severe with higher Fazekas scores in CAA controls (Table 3). Nevertheless, no item remained significant after adjustment for time from symptom onset to MRI except for the disseminated CSS (OR = 0.06 [0.00–0.47], adjusted *p* value = 0.0038). No difference was observed in terms of hippocampal atrophy.

**Table 3:** Radiological data compared to CAA controls

	***APP*** duplication with CAA on MRI	CAA comparison group	***Crude***		***After adjustment for onset-MRI delay***^***a***^	
	(***n*** = 14)	(***n*** = 40)	***OR/CI***	***p*** value	***OR/CI***	***p-value***
**Blood-sensitive sequence**						
- T2*	13	36				
**- SWI or SWAN**	1	4				
**Age at IRM (years, mean** ± **SD)**	55.2 ± 6.2	68.2 ± 8.5		9.2 × 10^−7^		
**Time from symptom onset to MRI (years, mean** ± **SD)**	4.1 ± 3.1	3.3 ± 3.6		0.458		
**Hemorrhages ICH and CSS**						
**Presence of ICH**						
**Lobar**	10 (71.4%)	24 (60.0%)	1.55 [0.36 ;8.02]	0.746	1.15 [0.20 ; 6.68]	0.873
**Posterior fossa**	2	0				
**Deep**	0	0				
**Presence of cortical superficial siderosis**						
**- At least one sulcus (focal and disseminated)**	2 (14.2%)	21 (52.5%)	0.15 [0.01;0.80]	**0.013**	0.33 [0.04;2.14]	0.266
**- Disseminated CSS only**	0	15 (37.5%)	0 [0.00;0.59]	**0.005**	0.06 [0.00;0.47]	**0.0038**
**Microbleeds**						
**Presence in posterior fossa**	6 (42.8%)	7 (17.5%)		0.07		
**Lobar MBs (mean** ± **SD)**	110.7 ± 146.6	56.7 ± 121.3	55.3 [− 146.8; 36.1]	0.221	47.5 [− 56.6; 51.8]	0.364
**Ratio post./ant.**	10.1 ± 12.5	5.3 ± 15.2	4.5 [− 13.0; 4.0]	0.291	8.1 [− 3.7; 20.0]	0175
**Presence in deep gray matter**	2 (14.2%)	0				
**Infarct**						
**Presence of lacunes**	0	5 (12.5%)		0.31		
**Presence of large vessel infarcts**	2 (14.2%)	1 (2.5%)		0.16		
**WM hyperintensities (Fazekas scale, mean** ± **SD)**						
**Modified Fazekas score in the pre-rolandic WM regions**	0.9 ± 0.64	1.6 ± 0.9	0.59 [0.13; 1.04]	**0.013**	− 0.60 [− 1.13; − 0.07]	0.125
**Modified Fazekas score in the post-rolandic WM regions**	1.5 ± 1.0	1.9 ± 0.9	0.41 [− 0.28; 1.10]	0.226	− 0.44 [− 1.08 ; 0.18]	0.262
**Hippocampal atrophy (Scheltens scale, mean)**						
**Right**	1.7 ± 1.4	1.6 ± 1.3	0.1 [− 1.1; 0.8]	0.780	0.10 [− 0.82; 1.02]	0.503
**Left**	2.0 ± 1.5	1.7 ± 1.2	0.25 [− 1.20; 0.70]	0.593	0.23 − 0.66; 1.13]	0.065

*CAA* cerebral amyloid angiopathy, *ICH* intra cerebral hemorrhage, *CMB* cerebral microbleed, *CSS* cortical superficial siderosis, *WM* white matter, *CI* confidence interval

^a^Linear (logistic) regressions were performed using each quantitative (resp. binary) variable of interest as output and group as explicative factor with adjustment for time from symptom onset to MRI. The last column gives *p*-values for the Dup/Non Dup group variable in each regression

### Neuropathological findings

Nine brain autopsies were available (BES_262, EXT_298, EXT_773, EXT_144, EXT_019, two patients from ROU_037 and two patients from EXT_028) including six previously reported (11, 15). Macroscopic examination revealed diffuse cerebral atrophy in all cases, which was more pronounced in the temporo-parietal regions with *a vacuo* ventricular dilatation as a consequence. Histologically, diffuse neuronal loss affecting cortical structures but also the brainstem was observed. Loss of Purkinje cells in the cerebellar cortex was variable.

Using tau and ubiquitin immunohistochemistry, numerous fibrillary tangles and senile plaques were observed in the hippocampal cortex, consistent with a definite diagnosis of AD with Braak & Braak stage V–VI [28] in all but one patient (EXT_144, presenting repeated ICH from the age of 42 and seizures but with no marked cognitive decline), who displayed no neurofibrillary tangles. Several neurofibrillary tangles were found in the thalamus, the putamen and the caudate nucleus in most of patients.

In all patients, amyloid plaques were present (as well as diffuse amyloid deposits), sometimes organized in rose petal-like formations in the cortical structures and predominating in the hippocampal formation and superficial cortex but moderate in the cerebellum. In one patient (ROU_037), multiple microcalcifications close to the amyloid angiopathy was observed within the cortical and subcortical occipital structures but no CT scan was available for this patient.

In all 9 cases, CAA was diffuse and severe with prominent thickening of leptomeningeal vessels, as well as superficial and deep intraparenchymatous small arteries, capillaries, and venules (Thal stages 3–5). Abundant circumferential amyloid deposits were found in the intima of arteriolar walls, with fragmentation of the internal elastic layer and media. CAA was also identified in the vessels of deep nuclei (EXT_773) in one patient and one recent hemorrhage was found in the pes pedunculi in another patient (EXT_144). In most of cases, ischemic changes and small infarcts were found near the amyloid vascular deposits. In the hemispheric white matter, microinfarcts were found close to the vascular amyloid deposits or at a short distance from affected vessels. Nearly all the meningeal vessels were strongly positive for Aβ40 antibodies within amyloid deposits, whereas Aβ42 immunoreactivity was detected on the core of amyloid plaques in the hippocampal and parahippocampal gyri. GFAP immunohistochemistry showed positive reactive astrocytes in several areas such as frontal and temporal lobes or cerebellum. Alpha-synuclein antibodies showed numerous structured LBs in the substantia nigra and locus coeruleus but also cortical structures in two patients, BES_262 [15] and EXT_773. Whenever performed, PrP antibodies were always negative in all structures studied.

## Discussion

In this study, we analyzed the clinical, radiological, and neuropathological features of 43 patients from 24 European families harboring an *APP* duplication, a rare cause of autosomal dominant AD and/or CAA, and compared their MRI features to those of 40 *APP*-negative CAA controls.

The wide range of different duplications shown here with distinct breakpoints (Fig. 3) and the diverse ethnicities of *APP* carriers reported in literature, as Japanese cases for instance [29], are not suggestive of a founder effect. Interestingly, one of our patients (EXT_773) harbored a de novo duplication. Given the reports of different-sized duplications and the identification of the first *APP* triplication, the *APP* locus appears to be a hotspot region for recombination, likely related to different regions with short tandem repeats [14]. In our study, 41 out of 43 patients were symptomatic patients and symptoms occurred between 42 and 63 years. Overall, 90.2% of symptomatic patients had major neurocognitive impairment, with a clinical diagnosis of amnestic AD and prominent episodic memory impairment in 41.4%, atypical presentation with prominent behavioral impairment in 9.7%, and severe dementia with quick bedridden state in 21.9% of patients. According to pedigrees, isolated cognitive decline occurred in 13/24 (54.2%) *APP* duplication families, with no reported ICH, whereas mixed presentation (cognitive decline in some affected relatives and ICH in others) occurred in 10 (41.6%) families. Mean age at onset of cognitive decline ranged from 42 to 60, highlighting the early onset and severity of cognitive impairment in patients harboring *APP* duplications and more than 90% of the cohort showed cognitive decline before 59.

Based on 16 *APP* duplication carriers, our group previously described seizures occurring in 31% of cases [16], with higher seizure risk compared to *PSEN1* or *APP* point mutation carriers in EOAD. An epileptiform activity consecutive to Aβ overproduction which modulates presynaptic and postsynaptic transmission in mice models was suggested, in addition to the effect of brain hemorrhages [30]. By adding 27 new carriers, our series further underlines the frequency of seizures in half of *APP* duplication carriers, independently of symptomatic ICH occurrence (in only 29.2%) or CSS on MRI (present in only 14.2%). Consequently, seizure occurrence in EOAD or CAA, whatever the clinical presentation, or in familial dementia, could be an argument for *APP* duplication screening.

Symptomatic ICH related to CAA occurred in 29.2% of our series, with a range at onset from 42 to 63 years, in line with the literature with a global rate of 30% when all reported *APP* duplications were gathered. In other autosomal dominant hereditary CAA such as Dutch type (HCHWA-D), Iowa or Italian *APP* point mutations, ICH occurred at similar ages (between 40 and 65 years) but with a higher prevalence of 75% in Iowa and Italian mutations [31] and up to 100% in HCHWA-D with fatal outcome in 2/3 cases after the first event [32]. Overall, 14/19 (73.6%) *APP* duplication carriers with available MRI fulfilled the radiological criteria for CAA, while only 5 displayed no hemorrhagic features. We identified several MRI features in those 14 carriers: scarcity of CSS, less extended white matter lesions, and high number of CMBs with prominent posterior location. Nevertheless, those results were not significant anymore after adjustment for time from onset to MRI except for the disseminated CSS (OR = 0.06 [0.00–0.47], adjusted *p* value = 0.0038). One obvious explanation may be the early age of onset of *APP* duplication carriers, but this may also suggest a correlation between *APP* duplications and radiological profile. The lack of power due to relatively small number of patients may have prevented those results to be significant in multivariate analysis. It has been shown that the *APOE* genotype has an impact on the MRI characteristics of CAA patients with a correlation between *APOE2* and CSS and *APOE4* and higher number of CMBs [33, 34]. The underlying mechanisms of CSS and CMBs are now considered to be different, if not opposite [35]. In *APP* duplication carriers, the pathogenic mechanism is the overproduction of Aβ [12]. This mechanism could lead to a suggestive radiological pattern including CMBs but no disseminated CSS and less white matter lesions. On the opposite, altered perivascular clearance of the peptide Aβ is often described in late-onset and sporadic cases of CAA. Indeed, a higher number of CMBs is known to be associated with severe amyloid load [36], which is consistent with the more severe amyloid deposits found in *APP* duplication compared to other *APP* point mutations [13]. On the other side, interestingly, CSS has been specifically linked to clearance defect in CAA [37].

Given the wide clinical and radiological heterogeneity, we sought a correlation between phenotype and duplication size. This question was initially raised with DS. Indeed, in line with the common sharing of an extra copy of the *APP* gene, located on chromosome 21 and its consequent overexpression [12], EOAD is highly penetrant in DS [38]. However, ICH is a strikingly rare feature in DS, despite the deposition of large amounts of amyloid plaques in the brain parenchyma and vessels. A recent histological study comparing 34 DS to 4 *APP* duplication carriers revealed prominent diffuse Aβ plaques throughout the cerebral cortex in DS, associated with CAA confined to leptomeningeal vessels conversely to *APP* duplication phenotypes which exhibited capillary and arterial intraparenchymatous CAA with fewer Aβ plaques [13]. This suggests that one or several duplicated genes in DS may provide partial protection against the pro-hemorrhagic effects of *APP* duplication [39] similarly to *BACE2* involvement in age at onset of dementia in DS [40]. Hence, we investigated whether any of the surrounding genes could account for the hemorrhagic features in *APP* duplications but found no correlation (Fig. 3). Moreover, the high heterogeneity even between the same family, carrying the same duplication, is a strong argument for assuming that this variability cannot be explained at the level of the duplication itself. The pro-hemorrhagic effects of *APP* duplications may more likely be modulated by more distant genes on chromosome 21 or other factors. The *APOE* genotype should also be considered as all patients carrying an *APOE2* allele present hemorrhagic features on MRI in line with previous data obtained from sporadic CAA patients [41].

Overall, numerous fibrillary tangles and senile plaques, observed in the hippocampal formation and the isocortex, consistent with a definite diagnosis of AD were observed in all patients but one (presenting recurrent ICH). CAA was diffuse and severe with thickening of the leptomeningeal vessels, as well as superficial and deep intraparenchymatous small arteries, capillaries and venules, and small infarcts often found near amyloid vascular deposits. Interestingly, LBs in the substantia nigra and locus coeruleus but also cortical structures were found in 2/9 (22.2%) carriers, one of them already reported [10]. The association of LB-pathology with AD-type pathology is however well recognized in autosomal dominant AD due to *APP*, *PSEN1*, or *PSEN2* mutations but also DS [42]. The present work underlines the need for *APP* duplication screening in families with a mixed phenotype of EOAD and LB dementia.

Finally, some clinical and radiological features of CAA due to *APP* duplications could be helpful to study sporadic CAA. HCHWA-D, due to a fully penetrant *APP* mutation, was previously considered to explore new MRI biomarkers in sporadic CAA diagnosis [43, 44]. More specifically, one additional hemorrhagic lesion (i.e., CSS) was added to the modified Boston criteria in 2010 [45]. Additional radiological features are still a matter of debate in a new revised version of the Boston criteria, as the possibility to consider hemorrhages in deep brain territories when associated with other lobar locations, or CSS severity [45]. In HCHWA-D, no deep CMBs have been reported. Nevertheless, we found 2 deep CMBs in *APP* duplication carriers, and more importantly one autopsy described vascular amyloid deposits in deep grey vascular structures, in the absence of lipohyalinosis. Taken together, these results support a revision of the radiological criteria integrating lobar/deep ratio of CMBs. Regarding CSF profile, all but one *APP* duplication carrier showed decreased Aβ42 levels, underlying the potential diagnostic value of this biomarker, as previously suggested from a sporadic CAA cohort by our group [46].

In conclusion, phenotypes associated with *APP* duplications are characterized by EOAD and/or CAA with overall symptomatic ICH representing 30%. More than 10% of carriers showed an atypical presentation such as isolated behavioral disorders or hallucinations suggestive of Lewy body disease and frequent early seizures. Subsequent APP overproduction leads to high amyloid burden, notably within cerebral vessels as demonstrated by the neuropathological data, the high number of CMBs, and the possible occurrence of CAA-related inflammation. Overall, we suggest *APP* duplication screening in all patients with CAA or AD onset before or equal to age 65 or early-onset family history.

## Supplementary Information

**Additional file 1: Supp Figure 1.** MRI scans of two *APP* duplication carriers showing deep microbleeds (orange arrows). A: EXT_814 carrying a 5.7Mb duplication and B: EXT_1516 carrying a 0.95Mb duplication.

**Additional file 2: Supp Figure 2.** MRI scans of three patients from the BES_262 family showing the large heterogeneity of cerebral imaging. A: MRI of 262-001; B: MRI of 262-003; C: MRI of 262-004.

## Data Availability

De-identified database and statistical analysis plan will be shared upon reasonable request for 2 years after publication.
