# Supplementary figures and images for: Phenotype and imaging features associated with APP duplications
Source: Alzheimers Res Ther. 2023 May 11;15:93. doi: 10.1186/s13195-023-01172-2 (PMC10173644; doi:10.1186/s13195-023-01172-2)

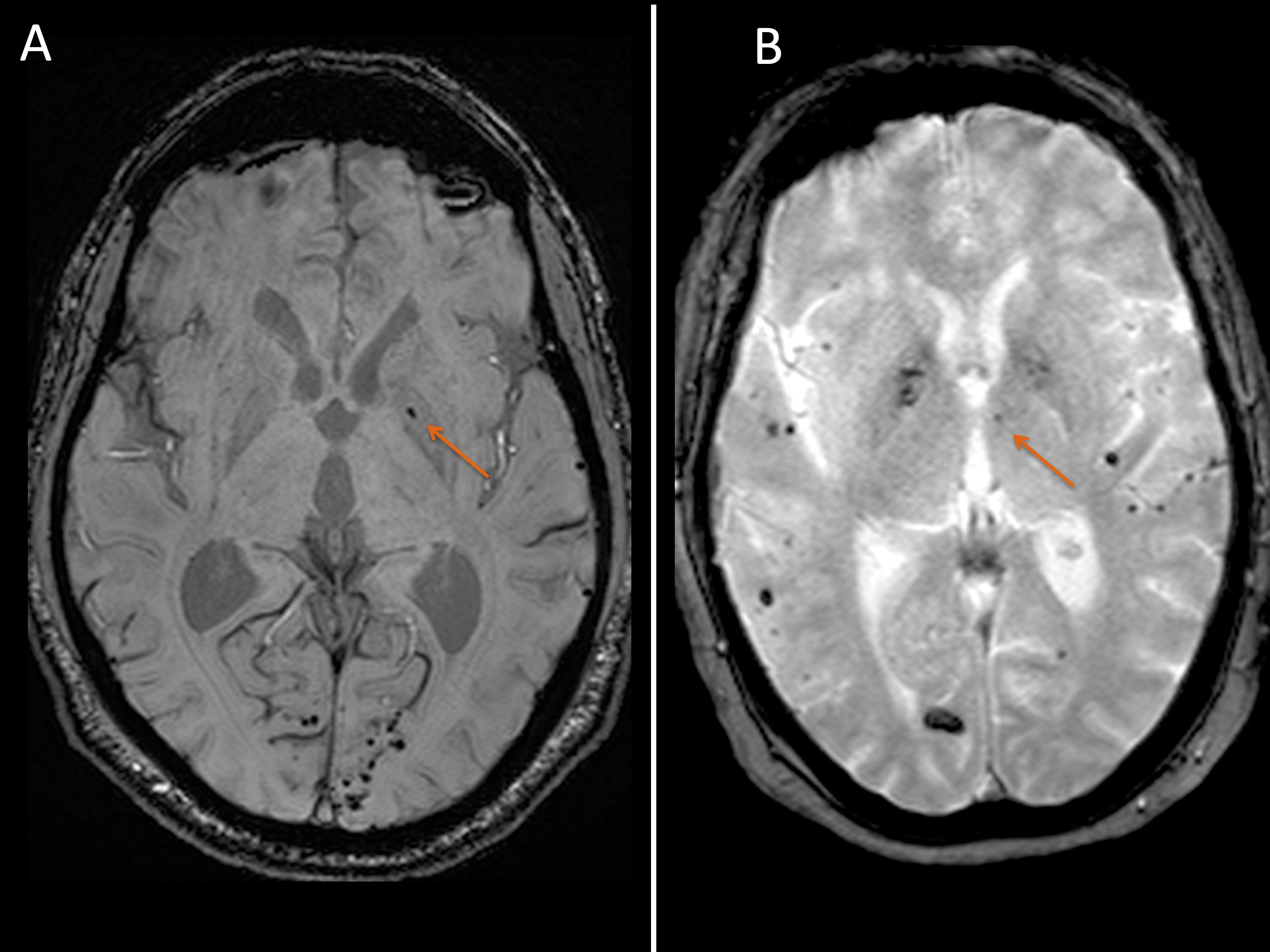

Supplement: Supplementary file 1 — Additional file 1: Supp Figure 1. MRI scans of two APP duplication carriers showing deep microbleeds (orange arrows). A: EXT_814 carrying a 5.7Mb duplication and B: EXT_1516 carrying a 0.95Mb duplication. [file 13195_2023_1172_MOESM1_ESM.jpg]

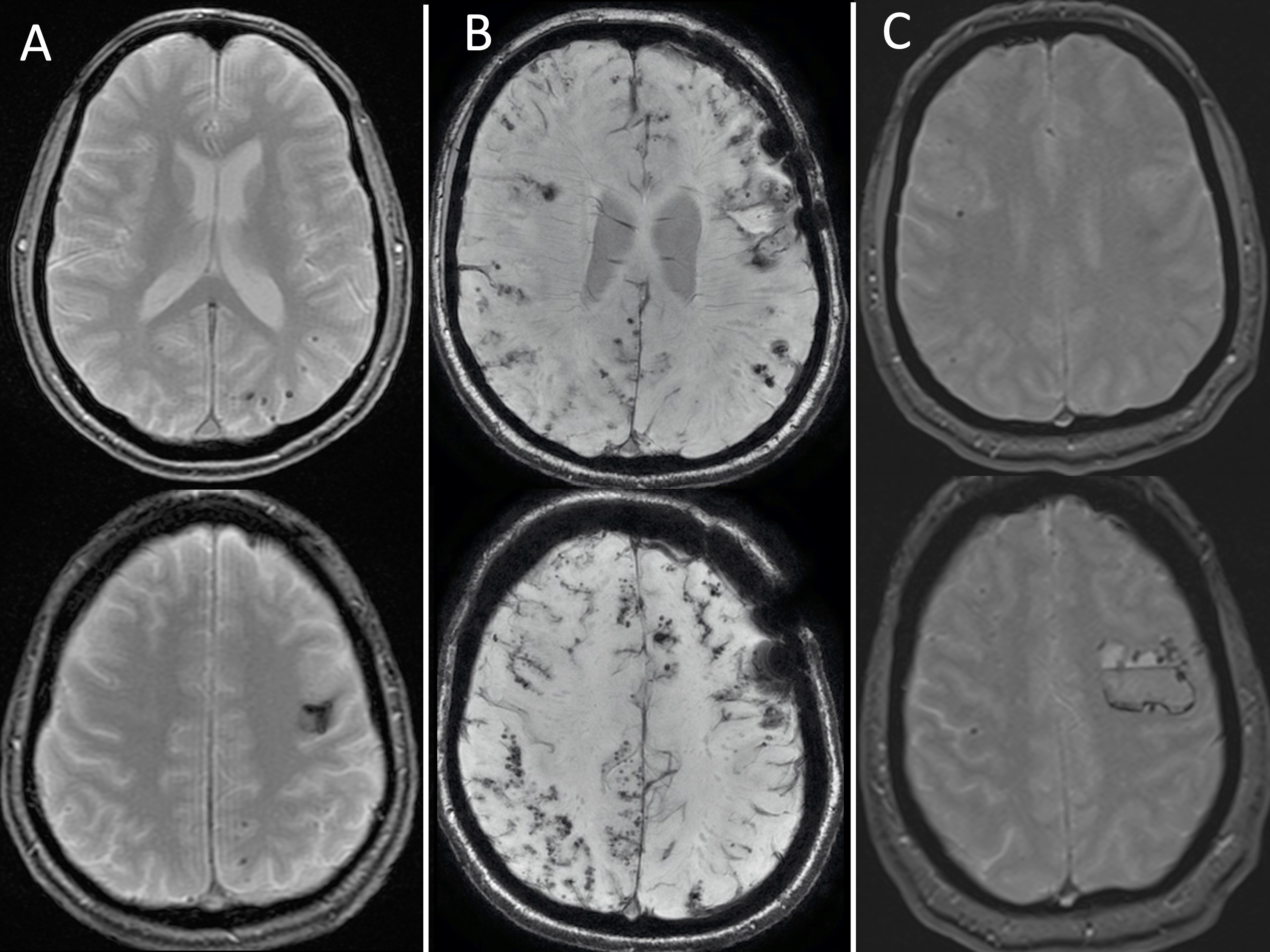

Supplement: Supplementary file 2 — Additional file 2: Supp Figure 2. MRI scans of three patients from the BES_262 family showing the large heterogeneity of cerebral imaging. A: MRI of 262-001; B: MRI of 262-003; C: MRI of 262-004. [file 13195_2023_1172_MOESM2_ESM.jpg]
